# How being a mother has made me a better family physician

**DOI:** 10.4102/safp.v68i1.6212

**Published:** 2026-01-16

**Authors:** Chantelle C. van der Bijl

**Affiliations:** 1 Department of Family Medicine, Faculty of Health Sciences, University of the Free State, Bloemfontein, South Africa

When I reflect on my first years as a family physician, I cannot separate my growth as a doctor from my journey as a mother. The two are deeply entwined; sometimes beautifully, sometimes painfully; but always truthfully. It is through becoming and being a mother that I have come to understand my patients in ways I never imagined as before even becoming a registrar. More than any textbook or Continuous Professional Development session, motherhood has taught me empathy, patience, humility, and the real meaning of care.

I had my first child right before my registrar time, and my second right in the middle of it. It was not a convenient time. Is there ever a convenient time to study while being a mother? There was the portfolio to fill, rotations to complete, and calls that did not pause for the pregnancy nausea or the aching fatigue of new motherhood. I returned to work with babies who barely slept and a heart that hurt with every handover to dad who did an amazing job at holding the fort while I brought home the bacon. I was determined to be good at both roles: doctor and mother; but I constantly felt like I was failing at one or the other.

Yet, something unexpected happened. The same rawness that made me feel so vulnerable also cracked me wide open to my patients. I began to see them differently: not as cases, but as people carrying invisible loads. When a mother brought in her sick toddler and could not remember when the fever started, I no longer assumed forgetfulness. I saw the sleepless nights, the worry, the mental fog of motherhood. When an elderly woman spoke slowly about her loneliness, I no longer rushed to the next point. I paused. I sat. I listened. It could be me some day.

## Motherhood has made me more human, more present

Motherhood has also brought me face to face with the limits of our healthcare system, and with my own powerlessness within it, even in private healthcare. I have sat in countless doctors’ rooms with my oldest child, waiting for an answer as to why she is always ill. I have felt the frustration of repeating my story to different clinicians, each kind but rushed. I have watched the clock, worried about making it to work on time. Finally, the answer came, Primary Immunodeficiency. And that is a story for another day. These experiences have given me a new sensitivity when I am on the other side of the desk. I now know that kindness is not a small thing. It is everything.

In my early years of training, I believed that knowledge was what made a good doctor. Now, I know that knowledge matters, but so does compassion, flexibility, and connection. I have stopped seeing the patient as the only ‘person in the room’ and started to see the whole context: the crying baby, the missed taxi, the unspoken fears. All those things they teach you in becoming a family physician; but I only truly understood it by being in the thick of it.

Being a mother has also deepened my commitment to continuity of care. I understand the value of relationships, of not needing to repeat yourself, of being known. When my own child sees their familiar doctor, I see the way their body relaxes. I want that for my patients too. To feel safe, remembered, and held over time. As family physicians, this is our unique strength. We do not just treat illness; we walk with people through life.

Of course, the balance is still hard. There are days when guilt knocks loudly. When I miss a sport match or concert for a clinic, or when my child gets the last crumbs of my energy. But I have also come to see that my children benefit from having a mother who is purposeful, who heals, who shows up for others. I hope that, in time, they will understand the privilege of this work.

Perhaps most importantly, motherhood has softened my ego. I have learned that I do not always know best. That sometimes the mother sitting in front of me knows more about her child than I do, even with all my degrees. I have learned to ask, ‘What do you think is going on?’ and mean it. I have learned to collaborate, not just instruct.

Upon reflection, I realise how my motherhood journey has shaped my early career as a family physician. It taught me to bring my whole self into the consultation, not just the doctor; but the woman who knows what it means to hold a feverish child, to run late, to be overwhelmed. I believe we need to normalise these life-stage experiences among registrars and early career family physicians. Parenthood, and all the growth that comes with it, is not a disruption to our professional identity. It is part of our complete self. And when we embrace that, we show up more fully for ourselves, our colleagues, and our patients.

In the next few years, I hope to keep growing into both roles, especially as a recently qualified family physician. I hope to be kinder to myself on the days when I drop the ball. I hope to keep bringing the lessons of motherhood into my practice: to lead with love, to listen deeply, to sit with uncertainty.

Being a mother has not made me a perfect doctor. But it has made me a better one.

